# Pamphlet Notices

**Published:** 1871-01

**Authors:** 


### Pamphlets.

*A Sketch of the Early History of Practical Anatomy.* An Introductory Address by Wm. W. Keen, M.D., Lecturer on Anatomy and Operative Surgery in the Philadelphia School of Anatomy, etc., etc. Published by the class. Philadelphia. 1870. Pp. 31.

In this address Dr. Keen has collected much curious and valuable information. Although Vesalius of the 16th century is the reputed father of anatomy, Herophilus and Erasistratus, 500 years before Christ, were zealous cultivators of the science of human anatomy, and are even charged with vivisections of human beings. Vesalius fled for his life on a similar charge, and lost his life by shipwreck in so doing.

Even in these days there is a vulgar idea that the profession indulge in a similar Parrhasian practice. The first public lecturer on anatomy in Alexandria, Clot Bey, 1700 years after Herophilus and Erasistratus, was stabbed with a poniard by a student when he first opened the thorax of a body. The blade slid over the ribs, and Clot Bey, perceiving that he was not seriously hurt, took a piece of plaster from his dressing-case, and, applying it to the wound, coolly observed to his class:

"We were speaking, gentlemen, of the disposition of the ribs and sternum, and I now have the opportunity of showing how a blow directed from above has so little chance of penetrating the thorax," and calmly went on with his lecture.

Fancy Prof. R. so situated.

The first dissections of modern times were made at Bologna, two centuries before Vesalius—but the custom soon fell into neglect. The first Monro says that in his student-days early in the last century his Scotch anatomy was limited to seeing the human body dissected once in two or three years. In the time of Sir Wm. Hunter, at the *end* of the same century, practical anatomy was unknown to the mass of the profession until he established his celebrated Great Windmill School.

As lately as 1866, Prof. Struthers, of Aberdeen, wrote: "Less than a generation ago, it was not an uncommon thing to find medical practitioners who had never dissected."

According to Dr. Keen's personal experience even now the majority of our students do not dissect the human body more than once. The slow progress of the practice, the legal restrictions and penalties, popular prejudices and resulting poverty of material and ingenious devices to procure it, are detailed, showing that human nature is much of the same pattern in all ages and countries. Hunter procured the body of the Irish giant, O'Brien, (whose skeleton, eight feet and four inches, is now in the Hunterian Museum) by bribing its guards, to the tune of £500, to permit him to steal it. Everybody has heard of Burke and his atrocities, which from that time even till now have caused the hairs of many a "loafer" to stand on end, but few are aware of the fact that this case led to a gradual improvement in the laws relative to the *subject*, until at last there is a remote approximation to justice and common sense in practice.

Not the least interesting portion of the address is that part which is devoted to the methods of preparing anatomical specimens, general and minute injections, preserving, embalming, etc., but space will not permit further notice at the present time. The lecturer has given us a very readable production, which we have no doubt his class fully appreciated.

*Annual Report of the Surgeon-General U. S. Army.* 1870.

This gives the annual statistics of the department. A few items will be of general interest:

"While the rate of mortality from wounds and injuries among the *colored troops* agrees with that among the white, their rate of mortality from disease is nearly double."

171 legs, 112 arms, 6 feet, and 12 apparatus, were furnished during the year.

The whole of the manuscript for the surgical volume of the first part of the Medical and Surgical History of the War, authorized by the Act of Congress, March 3, 1869, is now prepared, and a large amount of other valuable matter for the second volume of the Medical part is nearly printed.

A large and uniform increase of the collections in the Army

Medical Museum is reported. There are now 13,502 catalogued specimens. Great success has been attained in the microscopical section in the direction of photomicrography. The collection of crania includes 897 specimens, with 34 skeletons.

A liberal system of interchange of specimens with American and foreign institutions has been successfully organized.

There were 17,669 visitors registered during the year.

There are two vacancies as surgeons and 46 assistant surgeons; this has required the employment of contract surgeons, but it is thought better to have the vacancies filled.

In an accompanying pamphlet we find a series of extracts from letters, reviews and bibliographical notices of the publications of the Surgeon-General's office, all of which unite in expressions of high commendation of what has been done and is now being accomplished in this department.

We take especial pleasure in saying that in our opinion the work in progress, specimens of which have been from time to time made public, is one in itself of the very highest national importance, and which reflects the greatest credit upon the profession so ably represented by Surgeon-General Barnes and his excellent corps of assistants.

We repeat our former earnest hope, that no ill-timed and perverted ideas of contraction of expenditure, may prevent full and prompt publication of the inestimably valuable matter prepared and in course of preparation.

*Transactions of the New Hampshire Medical Society* (*Eighteenth Anniversary.*) Held at Concord, June 21 and 22, 1870.

This venerable Society still shows evidences of vigorous vitality. Several valuable papers are reported, among which we notice an interesting Medical History of New Hampshire, by A. B. Crosby, M.D.

We have marked several passages from this volume for reproduction in the JOURNAL.

